# Dementia in People with Severe/Profound Intellectual (And Multiple) Disabilities, and Its Natural History

**DOI:** 10.1080/19315864.2023.2240734

**Published:** 2023-07-27

**Authors:** Maureen B. G. Wissing, Johannes S.M. Hobbelen, Peter P. De Deyn, Aly Waninge, Alain D. Dekker

**Affiliations:** a Department of Neurology and Alzheimer Center, University of Groningen, University Medical Center Groningen, Groningen, The Netherlands; b Research Group Healthy Ageing, Allied Health Care and Nursing, Hanze University of Applied Sciences, Groningen, The Netherlands; c FAITH Research, Groningen, The Netherlands; d Academic Collaborative Center for PIMD, Groningen, The Netherlands; e Department of Practice-Oriented Scientific Research (PWO), Alliade, The Netherlands; f Department of General Practice & Elderly Care Medicine, University of Groningen, University Medical Center Groningen, Groningen, The Netherlands; g Laboratory of Neurochemistry and Behaviour, University of Antwerp; h Department of Neurology and Memory Clinic, Hospital Network Antwerp (ZNA) Middelheim and Hoge Beuken, Antwerp, Belgium; i Department of Health Psychology, University of Groningen, University Medical Center Groningen, Groningen, The Netherlands; j Royal Dutch Visio, Vries, The Netherlands

**Keywords:** Dementia, aging, intellectual disabilities, severe/profound intellectual (and multiple) disabilities, down syndrome, clinical records review

## Abstract

**Introduction:**

Although the prevalence of dementia increases among people with severe/profound intellectual (and multiple) disabilities (SPI(M)D), dementia in people with SPI(M)D is not yet fully understood. Therefore, this study aimed to characterize the natural history of dementia in people with SPI(M)D, in particular, the prevalence and time of onset of dementia symptoms.

**Methods:**

An explorative retrospective review of clinical records was conducted for people with SPI(M)D without dementia (*n* = 103), with questionable dementia (*n* = 19), and with diagnosed dementia (*n* = 19). Presence and time of onset of symptoms were extracted and compared between groups.

**Results:**

People with questionable dementia or diagnosed dementia had compared to people without dementia more symptoms regarding the cognitive, activities of daily living, behavioral/psychological, and motor domains. The most prevalent early symptoms were memory loss, declined walking skills, increased anxious, apathetic, and irritable behavior. Predictors for dementia were the number of cognitive, behavioral/psychological, and motor symptoms.

**Conclusion:**

These results contribute to enhance our understanding of dementia in people with SPI(M)D, which is essential for earlier recognizing and diagnosing dementia.

## INTRODUCTION

With aging, people are prone to develop age-related conditions such as dementia (Alzheimer’s Association, 2022). Dementia is the overarching term for a group of symptoms associated with a progressive decline in cognitive functioning from an individual’s previous level of functioning, which is severe enough to interfere with daily functioning (American Psychiatric Association, 2013; McKhann et al., 2011; World Health Organization, 2018). Dementia has several causes, Alzheimer’s disease (AD) being the most common one (Alzheimer’s Association, 2022). Recognizing and diagnosing dementia and its underlying etiology requires proper understanding of its natural history.

A vast number of studies have described which (early) dementia symptoms are generally observed among people with dementia in the general population (among others: Brodaty et al., 2015; Engelborghs et al., 2005; Giebel et al., 2021; Gilmore-Bykovskyi et al., 2020; Hendriks et al., 2022; Jost & Grossberg, 1996; Priefer & Robbins, 1997; Ramakers et al., 2007) as well as among people with intellectual disabilities (ID) and dementia, particularly those with Down syndrome (DS) (among others: Arvio & Bjelogrlic-Laakso, 2021; Benejam et al., 2020; Blok et al., 2017; Cosgrave et al., 2000; Dekker et al., 2015, 2018, 2021; Fonseca et al., 2020; Huxley et al., 2005; Moss & Patel, 1995; Nelson et al., 2001; Oliver et al., 1998; Temple & Konstantareas, 2005). DS is associated with an extremely high genetic risk of developing dementia due to AD (Ballard et al., 2016; Lott & Dierssen, 2010) and is actually considered according the International Working Group (IWG) 2 criteria as presymptomatic stage of AD (Dubois et al., 2014). While dementia research in people with ID is a growing field, only very few studies have focused on dementia in people with SPI(M)D (Wissing, Ulgiati, et al., 2022).

Dementia in people with SPI(M)D may differ from that in the general population and even from people with mild(er) ID. After all, already at baseline, people with SPI(M)D have severe/profound limitations in intellectual and adaptive functioning, i.e., conceptual, social, and practical skills (Schalock et al., 2021). They also often experience serious physical health problems, sensory impairments, and motor disabilities (Nakken & Vlaskamp, 2007; van Timmeren et al., 2017). Because of pre-existing severe/profound disabilities, people with SPI(M)D have not attained specific skills and often need lifelong support. As a result, never developed skills cannot alter and, therefore, cannot be considered as symptoms that may be indicative of dementia (Llewellyn, 2011; Sheehan, Sinai, et al., 2015). Moreover, dementia symptoms may be less noticeable in those with SPI(M)D: people with SPI(M)D have difficulty to self-report symptoms because their communication is limited and mainly non-verbal (Cooper & Smiley, 2007; Nakken & Vlaskamp, 2007). For the observation of symptoms, they thus depend on informants, such as family members and direct support professionals/caregivers (McKenzie et al., 2018).

The first studies to thoroughly identify practice-based observations of dementia symptoms in people with SPI(M)D have indicated which symptoms – aside from the pre-existing disabilities – are often observed by care professionals and family members (Dekker et al., 2021a; Wissing, Fokkens, et al., 2022). Although cognitive changes, e.g., memory loss, are main indicators for dementia in the general and mild ID population (Jamieson-Craig et al., 2010; World Health Organization, 2018), changes in activities of daily living (ADL) as well as behavioral and psychological changes were more prominent in people with SPI(M)D (Wissing, Dijkstra, et al., 2022). Such changes may indeed be indicative of dementia but could also be caused by – often treatable – conditions such as depression, delirium, vision problems, hearing problems, hypothyroidism, medication use, sleep apnea, or vitamin B12 deficiency (Moriconi et al., 2015; Scott & Barrett, 2007). Furthermore, such changes might relate to “normal” aging (Alzheimer’s Association, 2022). Correctly differentiating between, i.e., attributing changes to, dementia, comorbidities, or aging, is important to prevent over- and underdiagnosed dementia. Accurately diagnosing dementia requires thus a thorough process of ruling out other potential causes and proper understanding of differences between dementia and aging. However, very little research has examined observed differences between people with SPI(M)D with and without dementia (Wissing, Ulgiati, et al., 2022).

Increasing knowledge about dementia in people with SPI(M)D is necessary to improve recognition and diagnosis of dementia in early stages. Early identification of dementia allows to timely respond to a person’s changing wishes and needs by making informed choices (Dekker et al., 2021a; Janicki, 2011). Care can, for example, be tailored to the individual with SPI(M)D and dementia (Chapman et al., 2018; Dekker et al., 2021a). Moreover, diagnostic errors – missed, wrong or delayed diagnosis – as well as incorrect treatments can be avoided (Dekker et al., 2021a; Garcia et al., 1981). Early diagnosis also facilitates anticipation of the progression of dementia, for example, making choices about palliative care and end of life (Dekker et al., 2021a; Hughes et al., 2007; Roger, 2006).

To enhance understanding of dementia in people with SPI(M)D, this study aimed to characterize the natural history of dementia in people with SPI(M)D by determining the prevalence and time of onset of symptoms.

## METHODS

### Study Consortium

This study was part of a larger research project designed to identify dementia symptoms in people with SPI(M)D and develop a dedicated dementia screening instrument for people with SPI(M)D. The project “Practice-based questions about dementia in people with severe/profound intellectual (and multiple) disabilities” (Dekker et al., 2021a; Wissing, Dijkstra, et al., 2022; Wissing, Fokkens, et al., 2022; Wissing, Ulgiati, et al., 2022) is a collaborative effort of Hanze University of Applied Sciences, University of Groningen and University Medical Center Groningen (UMCG) with four Dutch care institutions spread across the country: Alliade, ‘s Heeren Loo, Ipse de Bruggen, and Royal Dutch Visio. In addition, data of people with SPI(M)D obtained within a similar study of clinical records in three Dutch care institutions Cosis, Philadelphia, and De Trans were used.

### Study Design

This study is an explorative retrospective analysis of clinical records of people with SPI(M)D. Different care institutions use different electronic clinical record systems. Nevertheless, for each participant, the same data were obtained from different components of clinical records, namely demographic information, physical examinations, diagnostic information, laboratory results, information about medication use, multidisciplinary consultations, psychological assessments, case notes drawn up by involved physicians, ID psychologists, and allied health care professionals.

### Ethics and Consent

The Medical Ethical Committee of the UMCG concluded that the Dutch Medical Research Human Subjects Act did not apply to this study (METc 2019/198). The study was registered in the UMCG Research Register (no. 201900193) and conducted in compliance with the UMCG Research Code and the EU General Data Protection Regulation. Legal representatives of people with SPI(M)D provided written informed consent for obtaining data from clinical records and processing/analyzing coded data for this study.

### Participants

Participants were purposefully recruited through the participating care institutions according the following inclusion criteria: severe, severe to profound or profound ID that originated before the age of 22, aged ≥40 years, with/without the presence of diagnosed syndromes (e.g., DS) or other disabilities (e.g., visual or motor impairments), with/without questionable dementia or diagnosed dementia. Participants were excluded from this study if no intellectual disability level was reported or when they had mild, mild to moderate, moderate, or moderate to severe ID. ID psychologists working within the care institutions were asked to identify eligible participants. Legal representatives of identified eligible participants received an information letter with informed consent forms. After providing informed consent, the intellectual disability level was checked before extracting data from clinical records.

### Data Collection

To extract data from clinical records, a data extraction form was developed in consultation with the project team, students (medicine, nursing, physiotherapy, and physician assistant), and care professionals working with people with SPI(M)D and experienced in keeping clinical records. The draft version of the data extraction form was pilot tested by extracting data from clinical records of 10 participants. The pilot allowed to improve the clarity and efficiency of the data extraction form. The ease of use was further optimized by constructing the data collection form in REDCap (Harris et al., 2009), hosted within the secured network of the UMCG.

The final version of the data extraction form consisted of two parts. The first part focused on participants’ characteristics, i.e., age, sex, living situation, attending day care, deaths, intellectual disability level, etiology of ID, a formal diagnosis of autism spectrum disorder, intelligence quotient, social-emotional functioning, baseline presence of verbal communication and walking skills. Additionally, information was collected about the presence of treated or untreated conditions – cerebrovascular accident, chronic pain, depression, delirium, epilepsy, hearing problems, hypothyroidism, sleep apnea, vision problems, vitamin B12 deficiency – which could cause dementia-like symptoms (Moriconi et al., 2015; Scott & Barrett, 2007). Furthermore, data were extracted about psychoactive medication use. Finally, data were collected about the presence of questionable dementia or diagnosed dementia, including information about the first year an individual was suspected of having dementia, the year of clinical diagnosis, and the etiology of dementia.

The second part focused on extracting data about the prevalence and time of onset of symptoms. According to diagnostic dementia criteria (American Psychiatric Association, 2013; McKhann et al., 2011; World Health Organization, 2018) and literature (Dekker et al., 2018, 2021; Ries, 2018; Strydom et al., 2010) symptoms were categorized into five domains: cognitive symptoms, ADL symptoms, behavioral and psychological symptoms, motor symptoms, and medical comorbidities. Each domain consisted of symptoms observed in people with SPI(M)D, which were obtained in one or more of the previous studies concerning dementia symptoms in SPI(M)D (Dekker et al., 2021a; Wissing, Fokkens, et al., 2022; Wissing, Ulgiati, et al., 2022). The total number of symptoms was 44, subdivided into 14 cognitive symptoms, 6 ADL symptoms, 11 behavioral and psychological symptoms, 10 motor symptoms, and 3 medical comorbidities.

The first domain contained 14 cognitive functions: awareness of proper order, judgment, language skills, losing objects, memory, object recognition, orientation in place, orientation in time, person recognition, planning, preference for (favorite) objects, problem solving, responsiveness, and understanding visual images/spatial relationships. Within the ADL domain, the six items concerned dressing, eating/drinking skills, grooming, showering/bathing, toilet use, and stair climbing. In the behavioral and psychological domain, the 11 items comprised aggressive, anxious, apathetic, depressive, disinhibited, eating/drinking, irritable, obstinate, psychotic, restless/stereotypic behavior, and sleeping problems. Motor functions were balance, choking, cramps, fall frequency, movement speed, muscle strength, stiffness, transfers/mobility, walking skills, and wheelchair use. Finally, epilepsy, incontinence, and weight formed the last domain.

Similar to the data extraction method of Jost and Grossberg (1996), we identified in clinical records the presence and time of onset of the symptoms. For each item, text fragments describing such an item, including the year in which a text fragment was written, were extracted from clinical records. If text fragments indicated a change, the item was coded as “presence of symptom.” Given that dementia is characterized by a decline in cognitive and ADL functioning (American Psychiatric Association, 2013; McKhann et al., 2011; World Health Organization, 2018), items within the cognitive and ADL domain were coded as “presence of symptom that decreased” (except for losing objects, which was coded as “presence of symptom that increased”). For behavioral and psychological symptoms, mainly an increase but also a decrease in frequency/severity of behavior may be observed (Dekker et al., 2018, 2021). Therefore, behavioral and psychological items were coded as either “presence of symptom that increased” or “presence of symptom that decreased.” Depending on the item, motor changes and changes in medical comorbidities were coded as “presence of symptom that decreased” or “presence of symptom that increased.” If the text fragments for a specific item indicated multiple changes, e.g., a decrease in 2018 and an increase in 2020, the first reported change was coded and added to the data extraction form. When text fragments did not comprise any indication of a change, the item was coded as “absence of symptom.” Lastly, if no text fragments for a particular item were identified, the code “not reported” was assigned to that item.

From June 2021 until September 2022, one researcher (MBGW) collected raw data and completed the data extraction form by coding the raw data for all participants, including the 10 clinical records of the pilot. Doubts about whether a symptom was absent or present were resolved in consultation with the project team. Two months after initial data extraction, the researcher coded identified item text fragments (in total 455) once more for a subset of 14 randomly selected participants, i.e., 10% of the total sample. The number of concordant codes was 429. Intracoder percent agreement, i.e., number of concordant codes/total number of identified item text fragments × 100 (Gisev et al., 2013), was 94.3%.

### Data Analysis

Extracted data were exported from REDCap to SPSS Statistics version 28 (IBM, Corp). Based on data about the absence/presence of questionable dementia or diagnosed dementia, participants were categorized into three groups: 1) SPI(M)D without dementia, 2) SPI(M)D with questionable dementia. i.e., the individual was suspected of having dementia but does not (yet) clearly meet the diagnostic criteria, and 3) SPI(M)D with clinically diagnosed dementia. For each group, participants’ characteristics were presented using descriptive statistics: chi-squared tests were used to compare categorical data and ANOVA to compare normally distributed continuous data (age) between groups.

To determine the prevalence and time of onset of dementia symptoms, we followed the analyzing method as described in the study by Jost and Grossberg (1996). Firstly, the prevalence, i.e., the proportion of individuals with diagnosed dementia exhibiting a symptom, was calculated for all identified symptoms. Secondly, the time of onset of symptoms was calculated by subtracting the year of diagnosis from the year at which the first change was reported. This could only be calculated if items were coded as “presence of symptom that decreased” or “presence of symptom that increased,” the year of diagnosis and year at which the first change was reported was known. Thereafter, the mean time of onset – separately for increase and decrease of a symptom – was calculated for each symptom. A time-density plot, in which the mean time of onset of a symptom was plotted against the prevalence of a symptom, was used to present results. Time zero represented the time of diagnosis. A negative time value indicated that the mean time of onset of a symptom was before the diagnosis and for a positive time value the mean time of onset of a symptom was after the diagnosis. In the plot also the mean time dementia was first suspected (year of diagnosis minus the first-year dementia was suspected) was displayed.

Furthermore, the prevalence of each symptom was also calculated for the group without dementia and the group with questionable dementia. Chi-squared tests were applied to identify differences in the prevalence between the three groups. When nothing was reported about an item in the clinical records, data were considered to be missing and thus not included in the analysis. Moreover, the number of symptoms per domain was calculated for each participant. Differences between the groups were compared using Kruskal–Wallis tests. Bonferroni–Dunn’s multiple comparisons post hoc tests were carried out when significant differences were found.

Finally, multinomial logistic regression, with the odds ratio (OR) being the main outcome measure, was used to analyze whether the number of symptoms per domain – cognitive, ADL, behavioral and psychological, motor, and medical comorbidities – could predict whether a person had questionable dementia or diagnosed dementia. The group which had no dementia was considered as reference category to which the other two groups were compared. Additionally, the analysis was performed again with the questionable dementia group as a reference category to also compare the questionable and diagnosed dementia group. For the statistical tests – except post hoc tests – a *p-*value <0.05 was considered significant.

## RESULTS

Legal representative of 266 identified eligible participants received an information letter with an informed consent form. Legal representatives of 168 eligible participants provided written informed consent, 19 did not provide consent, and 79 did not respond. Out of the 168 participants, 27 individuals were excluded based on exclusion criteria: no severe/profound ID (*n* = 14), intellectual disability level not reported (*n* = 4), deceased between consent and data extraction causing clinical records not to be accessible anymore (*n* = 9). The 141 included participants were grouped by the absence/presence of questionable dementia or diagnosed dementia: 103 had no dementia, 19 had questionable dementia, and 19 had diagnosed dementia.

### Participants’ Characteristics

All participants lived in residential facilities of care institutions and attended day care. Further participants’ characteristics are presented separately for each group in Table 1. What stands out in this table is that the presence of a syndrome significantly differed between groups (*p* < 0.001). Among the 103 persons without dementia, 9.7% had DS, whereas 52.6% and 63.2% had DS in the group with questionable and diagnosed dementia, respectively. Additionally, the baseline presence of walking skills differed significantly between groups (*p* = 0.009). All persons with questionable dementia and 94.7% with diagnosed dementia were at baseline able to walk while this was true for 80.6% in the group without dementia. No significant differences were found between groups concerning the prevalence of conditions that could cause dementia-like symptoms. Often nothing was reported about such conditions as chronic pain, sleep apnea, vitamin B12 deficiency, cerebrovascular accident and delirium in clinical records of those with questionable and diagnosed dementia. Lastly, the use of any psychoactive medication use (yes/no) (*p* = 0.040) and the total number of psychoactive medication used (*p* = 0.001) differed significantly between groups.

**Table 1.** Characteristics of three study groups.

**Participants’ characteristics**	**No dementia *n* = 103**	**Questionable dementia *n* = 19**		**Diagnosed dementia *n* = 19**		***p***
Age (years, mean ± SD (min.–max.))	64.6 ± 1.1 (43.0–89.0)	61.0 ± 8.9 (47.0–81.0)		65.6 ± 10.1 (48.0–85.0)		0.122
	49.5	36.8		36.8		0.408
Deaths among included participants (%)	12.6	15.8		15.8		0.890
Intellectual functioning: severe; severe/profound; profound (%)	56.3; 1.0; 42.7	68.4; 5.3; 26.3		52.6; 10.5; 36.8		0.195
Presence of syndrome: DS; other genetic syndrome; no/unknown (%)	9.7; 14.6; 75.7	52.6; 0.0; 47.4		63.2; 0.0; 36.8		<0.001*
Autism spectrum disorder: formal diagnosis; signs but no diagnosis (%)	14.6; 27.2	5.3; 21.1		0.0; 21.1		0.449
IQ-score available (%)	2.9	10.5		10.5		0.225
Social-emotional functioning: 0–6 months; 6–18 months; 18–36 months; 3–7 years; not reported (%)	19.4; 18.4; 5.8; 1.9; 54.5	10.5; 15.8; 5.3; 10.5; 57.9		15.8; 10.5; 15.8; 57.9		0.318
Verbal communication: able; no longer; never (%)	39.8; 2.9; 57.3	52.6; 0.0; 47.4		42.1; 5.3; 52.6		0.679
Walking skills: able; never (%)	80.6; 19.4	100.0; 0.0		94.7; 5.3		0.009*
**Conditions which could cause dementia-like symptoms**			**Not reported (%)**		**Not reported (%)**	
Vision problems: treated; untreated (%)	25.2; 62.1	36.8; 52.6	0.0	42.1; 57.9	0.0	0.162
Hearing problems: treated; untreated (%)	15.5; 38.8	21.1; 52.6	0.0	26.3; 52.6	0.0	0.257
Epilepsy: treated; untreated (%)	48.5; 10.7	15.8; 15.8	5.3	47.4; 5.3	0.0	0.051
Hypothyroidism: treated; untreated (%)	10.7; 0.0	15.8; 5.3	10.5	36.8; 0.0	10.5	0.022
Depression: treated; untreated (%)	3.9; 1.0	10.5; 0.0	36.8	5.3; 0.0	52.6	0.870
Chronic pain: treated; untreated (%)	4.9; 0.0	10.5; 5.3	73.7	1.5; 0.0	63.2	0.380
Sleep apnea: treated; untreated (%)	1.0; 1.9	0.0; 5.3	78.9	0.0; 0.0	94.7	0.831
Vitamin B12 deficiency: treated; untreated (%)	0.0; 0.0	0.0; 0.0	42.1	0.0; 0.0	36.8	-
CVA (%)	10.7	15.8	63.2	21.1	63.2	0.616
Delirium (%)	1.0	0.0	100.0	5.3	94.7	0.306
**Psychoactive medication use**						
Any psychoactive medication use (%)	72.8	47.4		63.2		0.040*
- Antiepileptics (N03A, %)	51.5	26.4		42.1		0.090
- Antipsychotics (N05A, %)	30.1	21.1		26.3		0.673
- Anxiolytics (N05B, %)	6.8	10.5		10.6		0.789
- Hypnotics and sedatives (N05C, %)	3.9	0.0		5.3		0.458
- Antidepressants (N06A, %)	14.6	10.5		15.8		0.862
- Antidementia (N06D, %)	0.0	0.0		0.0		-
- Opioids (N02A, %)	0.0	0.0		0.0		-
Number of psychoactive medications (% *n* = 0; 1; 2; 3; 4; not reported)	19.4; 29.1; 26.2; 14.6; 2.9; 7.8	47.4; 31.6; 5.3; 0.0; 10.5; 5.3		31.6; 36.8; 0.0; 15.8; 10.5; 5.3		0.001*

With respect to the prevalence of (un)treated conditions which could cause dementia-like symptoms, one would expect that these conditions were ruled out in the diagnostic process of dementia. Therefore, the proportion of individuals for whom nothing was reported about the prevalence of these (un)treated conditions are presented for both the group with questionable dementia and the group with diagnosed dementia. To compare differences between groups, ANOVA was used for normally distributed continuous data (age) and chi-squared tests were used for categorical data. Symbol: *p <0.05. Abbreviations: CVA, cerebrovascular accident; DS, Down syndrome; max., maximum; min., minimum; SD, standard deviation.

### Prevalence and Time of Onset of Symptoms

**Figure 1.**
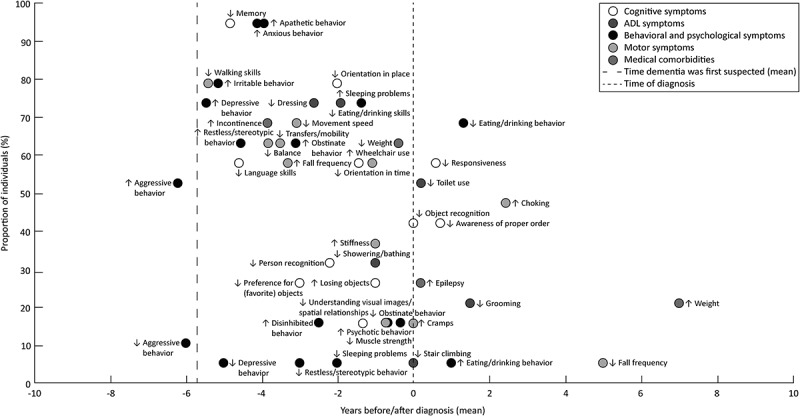
Time-density plot of dementia symptoms reported in clinical records of people with diagnosed dementia. The mean time of onset of a symptom is plotted against the prevalence of that symptom. Dashed lines represent the mean time dementia was first suspected −5.7 years before diagnosis – and the time of diagnosis, respectively. Symbols: ↓, decrease; ↑, increase. Abbreviation: ADL, activities of daily living.

The time-density plot (Figure 1) displays the mean time of onset and prevalence of dementia symptoms in those with diagnosed dementia. Among the 19 persons, 7 individuals with DS also had a diagnosis of AD, 3 for whom the cause of intellectual disability was unknown had a diagnosis of vascular dementia, and for the remaining 9 (5 with DS and 4 with an unknown cause of intellectual disability) no etiology of dementia was reported. The mean time between the first suspicions of dementia and the clinical diagnosis was 5.7 years (SD = 4.0, min.–max. = 0–16 years). Figure 1 shows that aggressive behavior – either an increase or decrease – was reported earlier than the mean time dementia was first suspected. Moreover, within the 5.7 years before the diagnosis, the most prevalent early reported symptoms (75–100%) were decreased memory, walking skills and increased anxious, apathetic, and irritable behavior. Decreased orientation in place was also prevalent in more than 75% of individuals with dementia, and was reported two years before the diagnosis. Furthermore, early symptoms with a prevalence between 50 and 75% were increased depressive, restless/stereotypic behavior, and decreased language skills. In the four to two years before the diagnosis, commonly reported symptoms (50–75%) were increased incontinence, fall frequency, obstinate behavior and decreased balance, transfers/mobility, movement speed, and dressing. Two years before diagnosis, there were five symptoms with a prevalence between 50% and 75%, namely decreased eating/drinking skills, orientation in time, weight and increased sleeping problems, and wheelchair use. Finally, decreased toilet use, responsiveness, and eating/drinking behavior were also prevalent symptoms in more than 50%, but were, generally, reported after the diagnosis of dementia.

### Differences in the Prevalence of Symptoms

**Figure 2.**
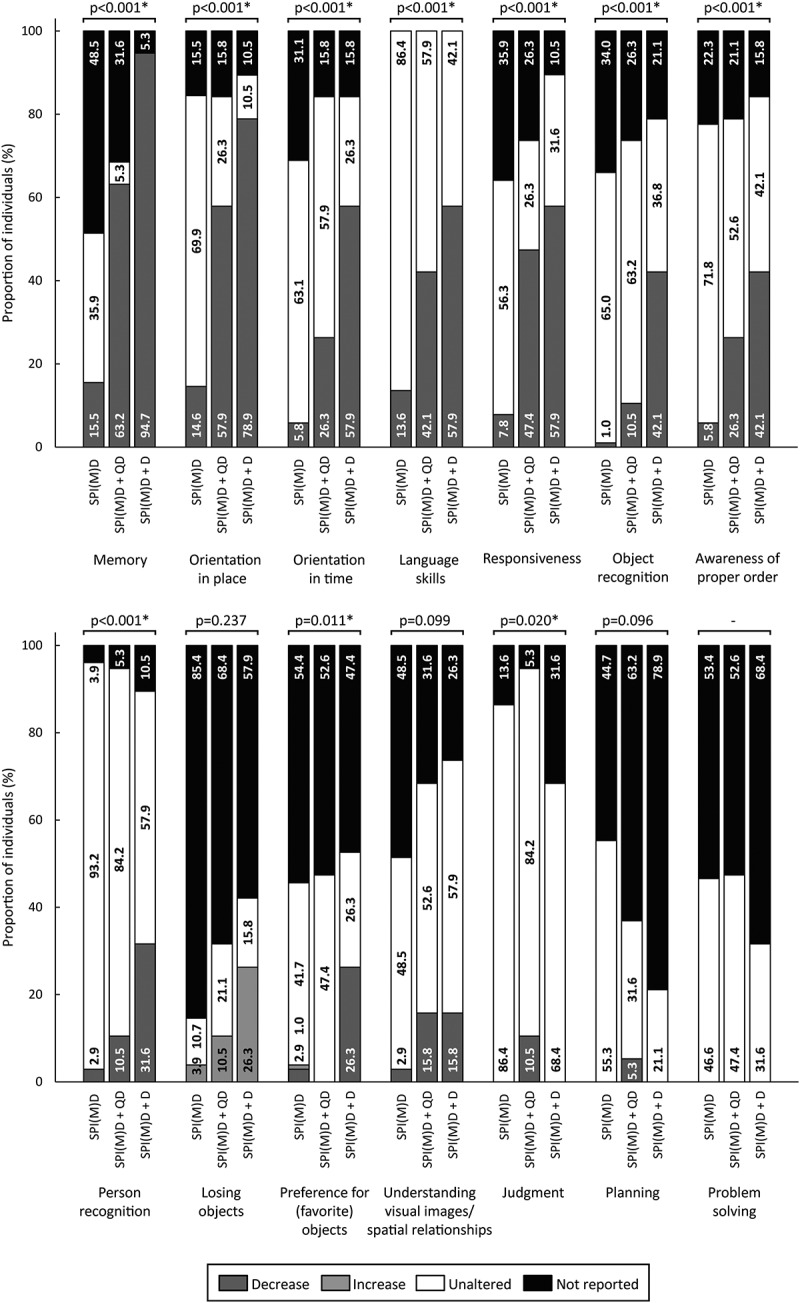
Prevalence of cognitive symptoms per group: no dementia (SPI(M)D), questionable dementia (SPI(M)D + QD) and diagnosed dementia (SPI(M)D + D). From left to right, symptoms are presented from most to least frequently reported for those with diagnosed dementia. Chi-squared tests were used to identify differences between groups. Symbol: *p <0.05. Abbreviation: SPI(M)D, severe/profound intellectual (and multiple) disabilities.

**Figure 3.**
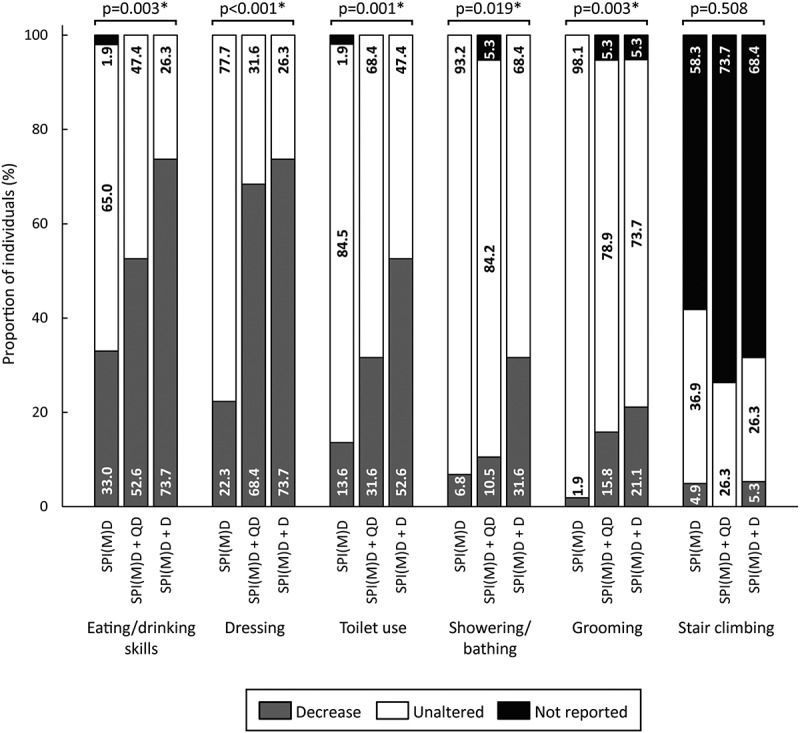
Prevalence of activities of daily living (ADL) symptoms per group: no dementia (SPI(M)D), questionable dementia (SPI(M)D + QD) and diagnosed dementia (SPI(M)D + D). From left to right, symptoms are presented from most to least frequently reported for those with diagnosed dementia. Chi-squared tests were used to identify differences between groups. Symbol: *p <0.05. Abbreviation: SPI(M)D, severe/profound intellectual (and multiple) disabilities.

**Figure 4.**
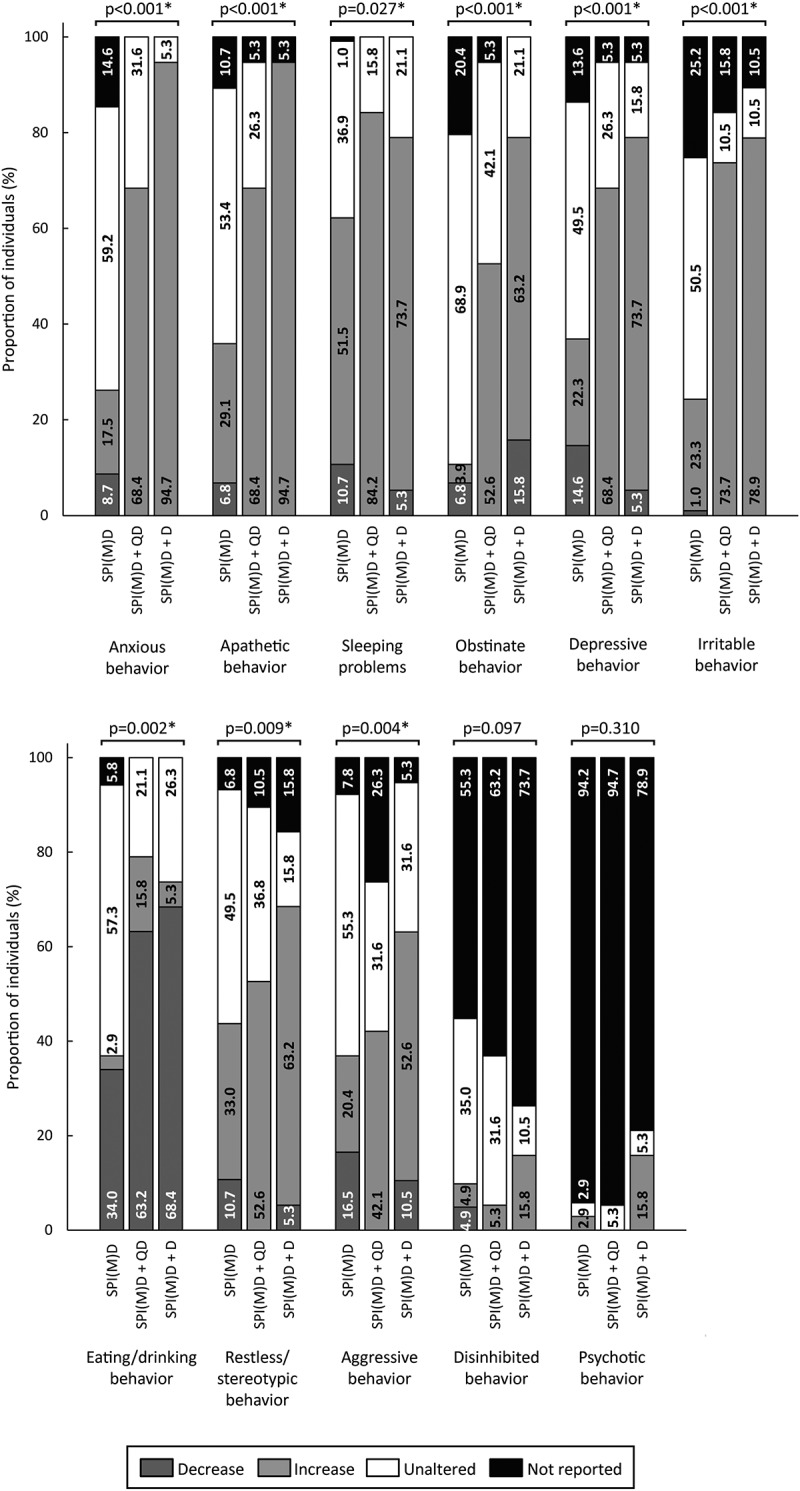
Prevalence of behavioral and psychological symptoms per group: no dementia (SPI(M)D), questionable dementia (SPI(M)D + QD) and diagnosed dementia (SPI(M)D + D). From left to right, symptoms – either decrease or increase – are presented from most to least frequently reported for those with diagnosed dementia. Chi-squared tests were used to identify differences between groups. Symbol: *p <0.05. Abbreviation: SPI(M)D, severe/profound intellectual (and multiple) disabilities.

**Figure 5.**
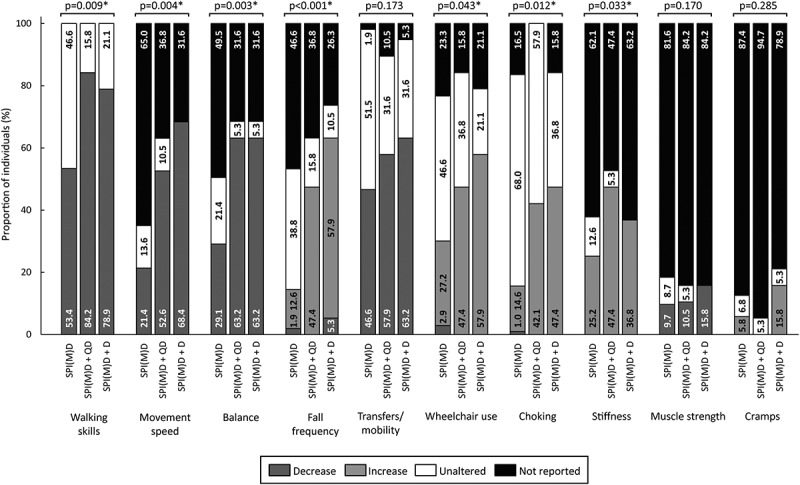
Prevalence of motor symptoms per group: no dementia (SPI(M)D), questionable dementia (SPI(M)D + QD) and diagnosed dementia (SPI(M)D + D). From left to right, symptoms – either decrease or increase – are presented from most to least frequently reported for those with diagnosed dementia. Chi-squared tests were used to identify differences between groups. Symbol: *p <0.05. Abbreviation: SPI(M)D, severe/profound intellectual (and multiple) disabilities.

**Figure 6.**
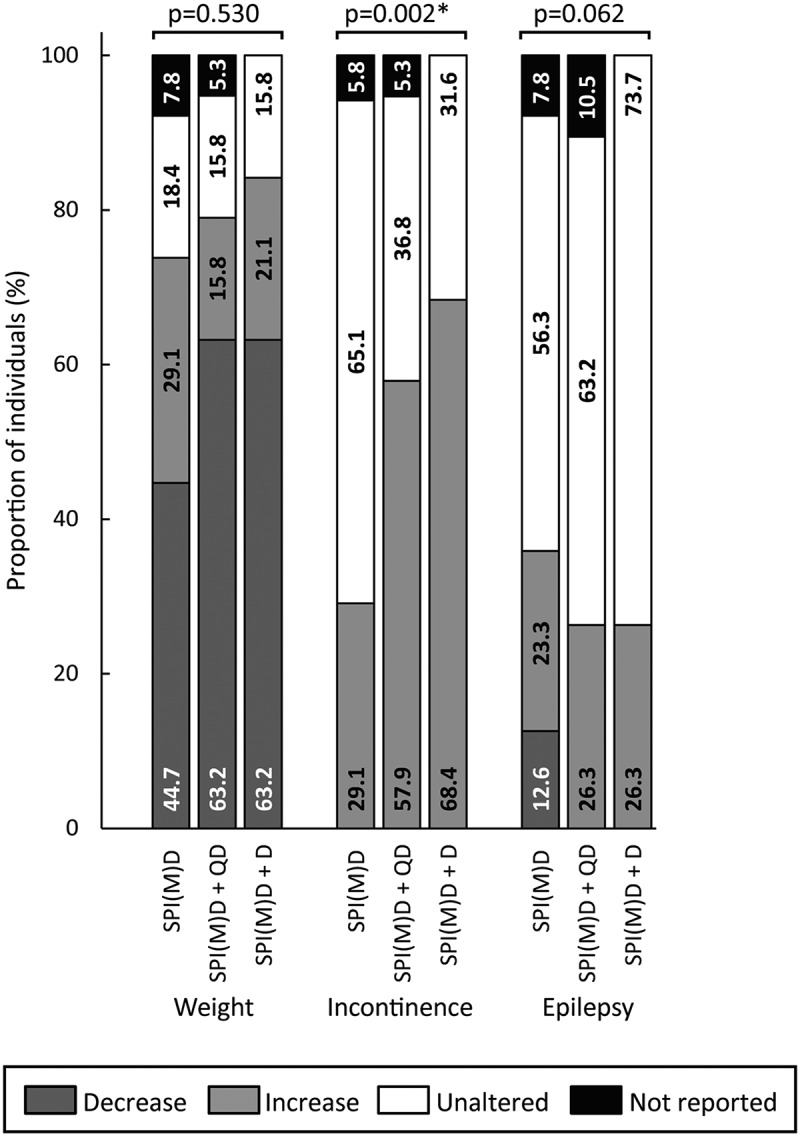
Prevalence of medical comorbidities per group: no dementia (SPI(M)D), questionable dementia (SPI(M)D + QD) and diagnosed dementia (SPI(M)D + D). From left to right, symptoms – either decrease or increase – are presented from most to least frequently reported for those with diagnosed dementia. Chi-squared tests were used to identify differences between groups. Symbol: *p <0.05. Abbreviation: SPI(M)D, severe/profound intellectual (and multiple) disabilities.

Figure 2–6 visualizes the prevalence of cognitive symptoms, ADL symptoms, behavioral and psychological symptoms, motor symptoms, and medical comorbidities per group: no dementia, questionable dementia, and diagnosed dementia. What stands out in these figures is that for the majority of symptoms the proportion of individuals exhibiting the symptom was lowest in the group without dementia, intermediate for those with questionable dementia and highest in the group with diagnosed dementia. Significant differences in the prevalence of symptoms between groups were found within all domains, which are explained in more detail in the subsequent paragraphs.

#### Cognitive Symptoms

Between groups, the following cognitive symptoms differed significantly: memory, orientation in place, orientation in time, language skills, responsiveness, object recognition, awareness of proper order, and person recognition (all *p*-values*<*0.001). For all eight symptoms, the proportion of individuals exhibiting the symptom was lowest in the group without dementia and highest in the group with diagnosed dementia. Preference for (favorite) objects differed significantly between groups as well (*p* = 0.011). Particularly, people with diagnosed dementia showed a decrease in preference for (favorite) objects. Regarding judgment, for which the groups also significantly differed (*p* = 0.020), only some persons with questionable dementia showed decreased judgment (Figure 2).

#### ADL Symptoms

ADL symptoms that differed significantly between groups (all *p*-values*<*0.05) were eating/drinking skills, dressing, toilet use, showering/bathing, and grooming. For these symptoms, the prevalence of a reported decrease was lowest in the group without dementia and highest in the group with diagnosed dementia (Figure 3).

#### Behavioral and Psychological Symptoms

A substantial number of behavioral and psychological symptoms were found to differ significantly between groups, namely anxious, apathetic, obstinate, depressive, irritable, restless/stereotypic, and aggressive behavior (all *p*-values*<*0.05). For these seven symptoms, the proportion of individuals exhibiting a symptom – either increase or decrease – was, again, lowest in the group without dementia and increased toward the group with diagnosed dementia. Sleeping problems (*p* = 0.027) as well as eating/drinking behavior (*p* = 0.002) significantly differed between groups. For these two symptoms, the prevalence was highest in the group with questionable dementia (Figure 4).

#### Motor Symptoms

Seven out of ten motor symptoms significantly differed between groups (all *p*-values*<*0.05): walking skills, movement speed, balance, fall frequency, wheelchair use, choking, and stiffness. For the symptoms movement speed, fall frequency, wheelchair use, and choking the prevalence was highest in the diagnosed dementia group, whereas for walking skills and stiffness the prevalence was highest in the group with questionable dementia. The prevalence for decreased balance was similar for the group with questionable and diagnosed dementia but lower in the group without dementia (Figure 5).

#### Medical Comorbidities

Concerning medical comorbidities, only incontinence differed significantly between groups (*p* = 0.002). The proportion of individuals showing increased incontinence was lowest in the group without dementia and highest in the group with diagnosed dementia (Figure 6).

### Differences in the Number of Symptoms per Domain

The number of symptoms for each domain are displayed per group in Table 2. Except for medical comorbidities, the number of symptoms per domain was lowest in the group without dementia and highest for those with diagnosed dementia. Significant differences between groups were found for cognitive, ADL, behavioral and psychological, and motor domain (all *p*-values*<*0.001). Post hoc tests showed that significantly more symptoms were reported for people with questionable dementia and people with diagnosed dementia compared to those without dementia (all *p*-values*<*0.05), whereas no differences were found between the group with questionable dementia and the group with diagnosed dementia.

**Table 2.** Number of symptoms per domain.

Domain	No dementia *n* = 103	Questionable dementia *n* = 19	Diagnosed dementia *n* = 19	*p*
Cognitive symptoms	0 (1), 0–7	3 (3), 0–8	6 (3), 2–8	<0.001*
ADL symptoms	1 (1), 0–5	2 (1), 0–5	2 (3), 0–6	<0.001*
Behavioral and psychological symptoms	3 (2), 0–9	6 (3), 2–9	8 (1), 4–10	<0.001*
Motor symptoms	2 (3), 0–7	4 (4), 0–9	6 (3), 0–9	<0.001*
Medical comorbidities	1 (1), 0–3	2 (1), 1–3	2 (2), 0–3	0.120

The number of symptoms for each domain (median (interquartile range), min.–max.) are presented separately for the groups. Kruskal-Wallis tests were used to compare differences between groups. Symbol: *p <0.05. Abbreviation: ADL, activities of daily living.

### Predictors of Dementia

The number of cognitive symptoms (OR = 2.12), behavioral and psychological symptoms (OR = 1.67), and motor symptoms (OR = 1.34) significantly predicted whether a person had questionable dementia versus no dementia (Table 3). The number of cognitive symptoms (OR = 3.92), behavioral and psychological symptoms (OR = 3.39), and motor symptoms (OR = 1.67) were also significant predictors for having diagnosed dementia versus no dementia. In both comparisons, the number of cognitive symptoms was associated with the highest risk for either questionable (OR = 2.12) or diagnosed dementia (OR = 3.92), followed by the number of behavioral and psychological symptoms (OR = 1.67 and 3.39, respectively). Lastly, people with questionable dementia were compared to those with diagnosed dementia. The number of cognitive symptoms (OR = 1.85) as well as the number of behavioral and psychological symptoms (OR = 2.03) significantly predicted whether a person had diagnosed dementia, but not the number of motor symptoms (Table 3).

**Table 3.** Predictive values of the number of symptoms per domain for the development of dementia.

Domain	No dementia versus questionable dementia^I^		No dementia versus diagnosed dementia^I^		Questionable versus diagnosed dementia^II^	
	OR (95% CI)	*p*	OR (95% CI)	*p*	OR (95% CI)	*p*
Cognitive symptoms	2.12 (1.38–3.26)	<0.001*	3.92 (2.05–7.51)	<0.001*	1.85 (1.08–3.17)	0.025*
ADL symptoms	0.67 (0.36–1.29)	0.240	0.44 (0.18–1.08)	0.730	0.65 (0.31–1.37)	0.260
Behavioral and psychological symptoms	1.67 (1.20–2.32)	0.002*	3.39 (1.61–7.16)	0.001*	2.03 (1.01–4.11)	0.048*
Motor symptoms	1.34 (1.01–1.78)	0.042*	1.67 (1.06–2.64)	0.028*	1.25 (0.82–1.90)	0.310
Medical comorbidities	0.89 (0.37–2.11)	0.790	0.46 (0.12–1.75)	0.250	0.52 (0.16–1.70)	0.280

Multinomial logistic regression was used to identify predictors for the development of dementia. Symbols: ^I^the reference category was no dementia; ^II^the reference category was questionable dementia; *p <0.05. Abbreviations: ADL, activities of daily living; CI, confidence intervals; OR, odds ratio.

## DISCUSSION

This explorative study aimed to characterize the natural history of dementia in people with SPI(M)D by determining the prevalence and time of onset of symptoms. Regarding the prevalence of symptoms, the results showed that the majority of symptoms were more frequently reported when people had questionable dementia and most prevalent when dementia was diagnosed. People with questionable dementia or diagnosed dementia had in total more cognitive, ADL, behavioral and psychological, and motor symptoms than those without dementia. With respect to the time of onset of symptoms, the results showed that the most frequent early symptoms were memory loss, declined walking skills, increased anxious, apathetic, and irritable behavior. The earliest symptom was aggressive behavior, for which mainly an increase but also a decrease in frequency/severity was reported. Before the diagnosis also changes in ADL, i.e., decreased dressing and eating/drinking skills, and medical comorbidities, i.e., increased incontinence and weight loss, were reported. The number of cognitive symptoms, behavioral and psychological symptoms, and motor symptoms were predictive for questionable dementia and for diagnosed dementia.

One of the earliest sign of dementia, particularly of AD, is memory loss (Alzheimer’s Association, 2022; Stern et al., 1993). This study confirms that memory loss also appeared in almost all persons with SPI(M)D and diagnosed dementia, on average already 4.8 years before the diagnosis. This result is likely to be related to the large number of people having DS, predisposed to develop AD with memory decline as a predominant symptom (Ballard et al., 2016; Lott & Dierssen, 2010). A decline in cognitive functions, such as memory can, however, also be part of “normal” aging (Alzheimer’s Association, 2022; Deary et al., 2009). Results showed that cognitive alterations were indeed observed in people without dementia as well, though cognitive symptoms were more common in those with questionable dementia or diagnosed dementia. In fact, results showed that cognitive symptoms were associated with a high risk on developing dementia, which is in line with findings in the general and the population with mild ID (Jamieson-Craig et al., 2010; Ramakers et al., 2007; World Health Organization, 2018). Overall, findings of this study thus indicate that alterations in cognitive functions – aside from pre-existing cognitive limitations – are indicative for dementia in people with SPI(M)D (Dekker et al., 2021a; Wissing, Fokkens, et al., 2022).

In all types of dementia, behavioral and psychological alterations are noticeable (Engelborghs et al., 2005; Finkel, 2000). The results of this study and previous studies demonstrated that behavioral and psychological changes are commonly observed in people with SPI(M)D and dementia (Dekker et al., 2021b; Wissing, Fokkens, et al., 2022). In line with findings in the general population (Hendriks et al., 2022; Ramakers et al., 2007), this study also demonstrated that behavioral changes are predictive for dementia specifically in people with SPI(M)D. Before diagnosis, increased anxious, apathetic, irritable, depressive and restless/stereotypic, obstinate behavior, and sleeping problems were reported. Moreover, these symptoms were also prevalent in more than half of the group with questionable dementia. Consistent with findings of dementia in people with DS (Dekker et al., 2018, 2021), this indicates that these symptoms are likely early “alarm signals” for dementia in people with SPI(M)D. The earliest dementia symptom was aggressive behavior, which is contrary to previous findings which showed that aggressive behavior was observed after AD diagnosis in the general population (Jost & Grossberg, 1996). A possible explanation for this might be that care professionals particularly report aggression earlier because aggressive behavior may be disturbing and harmful for the individual as well as their fellow residents, caregivers, and family members (Emerson, 2001; Jones & Kroese, 2007; Sheehan, Hassiotis, et al., 2015).

Not only cognitive symptoms and behavioral and psychological symptoms but also motor symptoms were found to be predictive for dementia in people with SPI(M)D. This finding is in agreement with previous research on dementia in the general population, reporting that gait disturbances predict dementia (Ramakers et al., 2007). Interestingly, in our study decline in walking skills was an early frequently reported motor symptom, which could only be observed if individuals are able to walk at baseline (Wissing, Fokkens, et al., 2022). The presence of baseline walking skills was higher in people with questionable dementia or diagnosed dementia than in those without dementia. It is likely that dementia is underrecognized and -diagnosed in people with SPI(M)D who have profound motor disabilities because certain symptoms like a decline in walking skills cannot be recognized within those persons (Dekker et al., 2021a; Wissing, Dijkstra, et al., 2022; Wissing, Fokkens, et al., 2022).

The number of ADL symptoms differed between those with/without questionable dementia or diagnosed dementia, but was no predictor. This might be related to the pre-existing limitations in the ability to perform ADL (Wissing, Dijkstra, et al., 2022; Wissing, Fokkens, et al., 2022), which vary among people with SPI(M)D (Nakken & Vlaskamp, 2007). Changes in ADL are less noticeable when people only perform small tasks within an ADL task and are not at all noticeable when someone fully dependent on others for performing ADL. Despite pre-existing limitations, ADL symptoms, like decline in eating/drinking skills, dressing, and toilet use were often reported in people with SPI(M)D and questionable dementia or diagnosed dementia. Previous dementia research in the general population described that ADL deteriorate particularly in later stages of dementia (Giebel et al., 2021; Marshall et al., 2012). In line with these findings, ADL symptoms are in those with SPI(M)D and diagnosed dementia generally later reported than cognitive symptoms, behavioral and psychological symptoms, and motor symptoms. This potentially also has to do with the tendency of caregivers and family members to provide more support if needed without being aware that the subtle decline in ADL functioning can be due to dementia.

Medical comorbidities were no predictor for dementia in people with SPI(M)D. Differences in medical comorbidities between people with/without questionable dementia or diagnosed dementia were only found for incontinence. This might be related to the fact that people with SPI(M)D already experience physical health problems like incontinence and epilepsy (Nakken & Vlaskamp, 2007; van Timmeren et al., 2017).

### Study Strengths

To the best of our knowledge, this explorative study is the first to extensively describe the prevalence and time of onset of dementia symptoms in people with SPI(M)D. This knowledge is of great essence to improve early recognition and diagnosis of dementia in people with SPI(M)D. Furthermore, a strength of this study is that not only the cognitive domain – main indicators for dementia in the general population and mild ID population (Jamieson-Craig et al., 2010; World Health Organization, 2018) – but also other domains, i.e., ADL, behavioral and psychological, motor domain and medical comorbidities were considered. To that end, we used previously found dementia symptoms in people with SPI(M)D (Dekker et al., 2021a; Wissing, Fokkens, et al., 2022; Wissing, Ulgiati, et al., 2022). Lastly, a strength is the use of the time-density plot, which is a convenient method of demonstrating both the prevalence of symptoms as well as the onset of symptoms over time in people with dementia (Jost & Grossberg, 1996).

### Study Limitations

Extracting data from clinical records allowed to identify if and when symptoms were reported. However, reliance on clinical records can also be considered as a limitation since reports could be incomplete: symptoms might have been observed but not reported or were underrecognized and thus not reported. Especially since knowledge about dementia in people with SPI(M)D is limited, it is conceivable that care professionals are often not educated sufficiently to recognize all symptoms, especially not in early stages. Moreover, documentation varied among care institutions. For example, certain institutions extensively reported on motor skills, whereas others reported on different prioritized areas. This may have resulted in underreporting symptoms in less prioritized areas. Altogether, these limitations may have caused an underestimation of the prevalence of symptoms. Another limitation of this study is that no second researcher independently extracted and coded data from (part of) the clinical records, and thus intercoder percent agreement could not be determined. Furthermore, a limitation was that the sizes of the subgroups were rather unequal. In each of the two groups of interest, i.e., questionable dementia and diagnosed dementia, 19 participants were included. The small subgroup sizes seem to be related to the complexity of recognizing and diagnosing dementia in people with SPI(M)D. Until recently, hardly any literature about dementia in people with SPI(M)D was available, and therefore it is very likely that dementia is underdiagnosed in people with SPI(M)D (Wissing, Ulgiati, et al., 2022). Despite the small subgroup sizes, the results of this study contribute to a better understanding of the natural history of dementia in people with SPI(M)D, which is essential to (earlier) recognize and diagnose dementia. Reducing underdiagnosis of dementia would allow future studies to more easily include larger and more equally divided study groups. Moreover, with a larger sample size also similarities and differences in the natural history of dementia in those with SPI(M)D with and without DS could be examined.

### Future Implications

Due to the complexity of recognizing and diagnosing dementia, dementia is diagnosed later in people with SPI(M)D (5.7 years after dementia was first suspected) compared to the general population (2.7 years after the onset of symptoms (Jost & Grossberg, 1995)). Earlier diagnosing dementia, and thus preventing delayed or underdiagnosed dementia, requires a diagnostic procedure dedicated to people with SPI(M)D. First of all, conditions causing dementia-like symptoms should be more thoroughly ruled out (differential diagnosis). This study showed that often nothing was reported about the presence of such treated and untreated conditions in people with questionable dementia or diagnosed dementia. Enhancing knowledge and understanding about dementia in people with SPI(M)D would also significantly benefit the process of diagnosing dementia. To facilitate the diagnostic process, dementia-related changes should be systematically identified and monitored by, for example, using a dementia screening instrument. However, today no such standardized instrument exists for people with SPI(M)D (Wissing, Dijkstra, et al., 2022). Therefore, there is a need to develop a tool to aid the diagnosis of dementia in people with SPI(M)D. Additionally, videotaping could potentially become a standard part of dementia diagnostics in people with SPI(M)D because it allows to examine the onset and progression of subtle dementia symptoms in more detail. Overall, steps toward an early diagnostic process and thus early diagnosis of dementia in people with SPI(M)D can only be undertaken when symptoms are recognized early by informants, such as family members and caregivers. Therefore, it is essential to develop training products about dementia in people with SPI(M)D to increase informants’ knowledge.

## CONCLUSION

This explorative study focused on the natural history of dementia in people with SPI(M)D. Presence and time of onset of symptoms were extracted from clinical records of purposefully selected participants with SPI(M)D with/without questionable dementia or diagnosed dementia. Differences in the prevalence of symptoms were found between those with/without questionable dementia or diagnosed dementia. Most symptoms were more common in people with questionable dementia and most prevalent in those with diagnosed dementia. People with questionable dementia or diagnosed dementia showed compared to those without dementia more cognitive, ADL, behavioral and psychological, and motor symptoms. Regarding the time of onset, memory loss, declined walking skills and increased anxious, apathetic, and irritable behavior were found to be early signs of dementia, present in almost all people with diagnosed dementia. The number of cognitive symptoms, behavioral and psychological symptoms, and motor symptoms were predictors for questionable dementia and diagnosed dementia. Together these results provide important insight into the natural history of dementia in people with SPI(M)D, which is essential to early recognize and diagnose dementia in people with SPI(M)D.
